# Development of Chlamydial Type III Secretion System Inhibitors for Suppression of Acute and Chronic Forms of Chlamydial Infection 

**Published:** 2012

**Authors:** N.A. Zigangirova, E.S. Zayakin, L.N. Kapotina, E.A. Kost, L.V. Didenko, D.Y. Davydova, J.P. Rumyanceva, A.L. Gintsburg

**Affiliations:** Gamaleya Research Institute of Epidemiology and Microbiology

**Keywords:** thiohydrazones, thiohydrazides, thiadiazines, type III secretion system, citotoxicity, Chlamydia, inhibitors, microscopy, electron microscop, morphology

## Abstract

The Type III secretion system (T3SS) is currently considered to be one of the main pathogenicity factors in Gram-negative bacteria, which exhibit different types of parasitizing activity. The presence of this structure is essential for the development of an acute infection; the chronicity of the infection is fundamentally dependent upon its functioning. In this regard, T3TS is one of the most promising targets for the development of broad-spectrum antimicrobial drugs that do not develop resistance and are efficacious for the acute and chronic forms of infection. The mechanism of action in drug development is based on the specific inhibition of T3SS, which should interrupt the infectious process, thereby enabling the immune system to eliminate the pathogen. As a result of pilot screening using specific cellular and bacterial tests, followed by chemical optimization and detailed characterization of the biological activity, a new class of chlamydial T3SS inhibitors was obtained. The selected compounds have obvious advantages over the currently available inhibitors of T3SS pathogens thanks to the high inhibitory activity of these compounds with minimal damaging effects on eukaryotic cells. Preclinical trials of the selected inhibitors are currently under way.

## INTRODUCTION 

Chlamydia are Gram-negative bacteria that parasitize intercellularly. T wo species of Chlamydia are common pathogenic agents responsible for diseases in humans. The Chlamydia infection caused by *Chlamydia trachomatis* is the most prevalent among sexually transmitted diseases,causing over 100 million new cases of the disease annually [1]. According to the WHO, the number of people in the world infected with Chlamydia by the most conservative estimate has reached a billion; the number of infections is on a steady increase even in the developed world. The fraction of cases of respiratory chlamydiosis caused by *C. pneumoniae * in the total structure of pneumonias stands at 20%; epidemic outbursts of this infection occur in European countries every 4–7 years (according to WHO data). As a result, up to 80% of the world population are infected with respiratory chlamydiosis during their lives. Chronic chlamydioses pose the most serious problem; it is a proven fact that these diseases act as a mechanism triggering severe chronic diseases, such as asthma, atherosclerosis, arthritis, female and male infertility, as well as pregnancy pathologies [2, 3]. 

The medical and socio-economic significance of searching for new-generation drugs using target-specific technologies is rooted in the absence of efficacious agents that can help treat chromic bacterial infections and the rapid development of pathogen resistance to the antibacterial agents used to treat acute infectious processes [4–6]. In the case of antibacterial drugs, this technology includes selecting the proteins responsible for the exhibition of pathogenic properties by the microorganism as targets; the subsequent search for specific inhibitors using computer software, organic synthesis techniques and experimental assaying; and verification of the predicted biological activity on model systems for the infectious process. 

Secretion of pathogenic factors (the proteins responsible for the exhibition of pathogenic properties by bacteria) into the macroorganism’s cell is the key mechanism underlying the development of an infectious process. A total of seven secretion systems, characterized by various specificities with respect to the molecules secreted and differences in the structure of the secretory apparatus, have been described thus far. One of these systems (referred to as the type III secretion system (T3SS)) transfers protein pathogenic factors from the bacterial cell directly into the cytoplasm of the eukaryotic cell. This “molecular syringe” has been detected only in pathogenic bacteria, since it is through its functioning that the bacteria with various types of parasitizing actions, exo- and endoparasites, exhibit their pathogenic properties [7]. Because of the conservative nature of this structure, in the taxonomically distant microorganisms that are behind socially significant infections, such as *Chlamydia, Salmonella, Shigella, Pseudomonas* , *Escherichia* , *Yersinia, Brucella, * etc *.* , it is reasonable to expect antibacterial drugs based on specific T3SS inhibitors to have a wide range of effects. 

In intracellular pathogens (Chlamydia being a typical example of such organisms), the transport system makes it possible to use the regulatory pathways of a host eukaryotic cell and to subsequently suppress the cellular response. T3SS is required at each stage of Chlamydial life cycle; it provides the possibility of intracellular reproduction of the pathogen upon both acute and chronic forms of the infection. T3SS inhibition results in the suppression of the *in vitro* reproduction of Chlamydia [8]. 

Several T3SS inhibitors, low-molecular weight compounds of different classes, which were selected by high-efficiency screening of chemical compound databases, have been discoveredthus far [9–13]. A substantial drawback of these compounds is their poor solubility in organic solvents and water. Moreover, these inhibitors exhibit significant toxicity towards mammal cells, resulting in the death of up to 60% of the cells in the presence of a specific inhibitory concentration (50 µM), a factor which restricts the development of these compounds for further application as antibacterial drugs. 

This study was aimed at designing pharmacologically promising compounds that can suppress acute and chronic infections which inhibit the secretion of chlamydial pathogenic factors, but do not have the aforementioned drawbacks. 

## METHODS 

### Bacterial strains and cell lines 

Reference strain of *C. trachomatis * BU-434 serovar L2 (АТСС VR 902B), * C. muridarum * strain Nigg(АТСС VR-123), and *C. pneumoniae* strain K-6 kindly provided by P. Saikkii (Finland), and the McCoy В cell line (a hybrid cell line consisting of human synovial cells and mouse fibroblasts) were used in this study. 

### Assessment of the toxicity for eukaryotic cells 

96- and 24-well plates and a one-day cell monolayer were used in the study. The cells were cultured for 24 h in the presence of various doses of inhibitors. The cytotoxic effect of the agents was assessed using three conventional procedures: methylene blue staining, the MTT assay (“Sigma”), and the calcein assay (LIVE/DEAD Viability/Cytotoxicity Kit for mammalian cells, Invitrogen, United States). 

### Cell infection with Chlamydia strains 

The МсСоу В cells were infected with Chlamydia with multiplicity of infection of 1 (MOI of 1) according to the conventional procedure [3]. 

### Immunofluorescent detection of Chlamydia development 

The intracellular chlamydial inclusions were detected via direct immunofluorescence (DIF) using monoclonal species-specific antibodies against the major outer membrane protein (MOMP) of *C. trachomatis * and fluorescein isothiocyanate (FITC) labelled genus-specific anti-chlamydial LPS antibodies (OOO Niarmedic Plus, Moscow). 

### Assessment of chlamydial progeny 

Chlamydial progeny was assessed via a semi-quantitative analysis based on immunofluorescence. Lysates of the infected cells were seeded onto a new cell monolayer. For this purpose, the 48-hour monolayer of infected cells was removed by a sucrose phosphate glutamine buffer (SPG) and lysed by freezing. The necessary lysate dilutions were prepared and then seeded onto a new monolayer. The cells were incubated for 48 h, fixed, and stained with FITC-labelled monoclonal antibodies for subsequent assessment of the results via luminescent microscopy. The count of infected cells was determined in 10 random locations within the visual fields; the average number of inclusion-forming units (IFU) per 1 ml of the specimen was calculated (the results of three independent experiments were used). 

### Detection of the *C. trachomatis* effector protein IncA  

A one-day monolayer of McCoy cells was infected with *C. trаchomatis* with MOI of 5. The compounds under study were added at varying doses eight hours post infection (the onset of the effector protein translocation to the inclusion membrane). After 24 h of incubation the cells were stained with primary anti-IncA-antibodies (Innovagen, Sweden) and secondary FITC-labelled antibodies. The cells were simultaneously stained with anti- *C. trachomatis* МОМР monoclonal antibodies. 

### Transmission electron microscopy (TEM) 

**Fig. 1:**
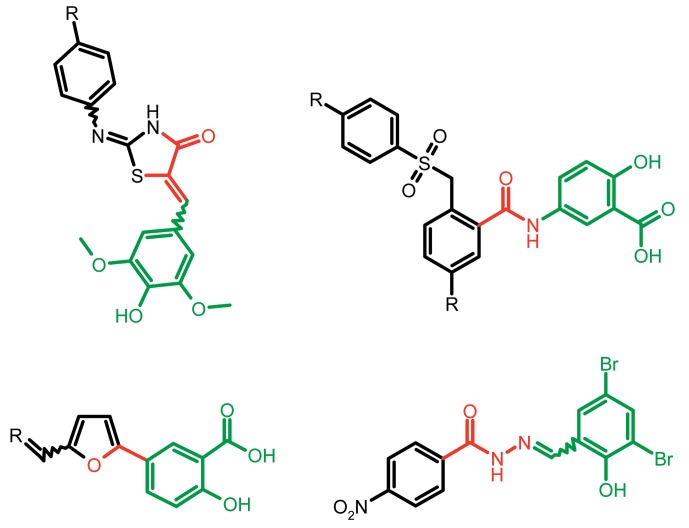
Structures of various classes of the known T3SS inhibitors.

**Fig. 2:**
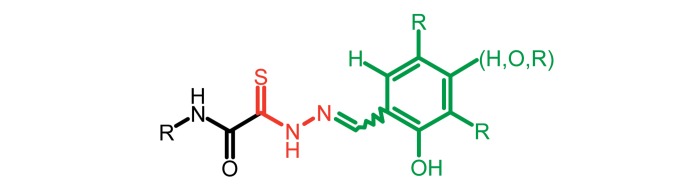
The general formula of thiohydrazones of the thiohydrazides of oxamine acids.

The cells were cultured and infected in 6-well plates. The cell pellet obtained via centrifugation for 10 min at 1500 rpm (Rotanta 460R, Hettich) was fixed in aIto-Karnovsky fixative. Post-fixation with OsO _4 _ and contrasting in aqueous uranyl acetate were used for this purpose. The specimens were subsequently dehydrated in an ascending series of alcohols, infiltrated in a 1 : 1 mixture of a LR White resin and 100% ethanol for 1 h and in a pure resin for 12 h at +4°С. Resin polymerization was performed at +56°С for 24 h. Ultrathin sections were then prepared, then they were contrasted with a lead solution (Reynolds) and analyzed using TEM Jeol 100B. 

### RNA isolation and analysis of gene expression 

RNA was isolated from the cell culture using the Trizol reagent (Invitrogen) 24 h post infection. The concentration of RNA pretreated with DNAse I (DNA-free™, “Ambion”) was determined on a NanoDrop ND-100 spectrophotometer (ThermoFisher Scientific, United States). The reverse transcription (RT) reaction was carried out using the “Reverse Transcription System” kit (Promega, United States). 

Real-time PCR with the resulting cDNA was carried out using primers to the following genes: *16S* rRNA (primer forward 5-’GGCGTATTTGGGCAT CCGAGTAACG, primer reverse 5’-TCAAATCCAGCGGGTATTAACCGCCT, Pb 5’-R6G-TGG CGG CCA ATC TCT CAA TCC GCC TAG A-BHQ2), *trpA* (primer forward 5’-CGG GAA TAA ATG GTG TGT GCG T, primer reverse 5’-TAAAGACATCCGTTCCGGCGTT, Pb 5’-ROX-ATC TTC CAG CAC CTT TAT CAC ACG GAG A-BHQ2), *incA * (primer forward 5’-CTA CAG AAG AAA TGC GCA AAC TTT, primer reverse 5’-AAT GAT TGC TGG TTA TGC GCT AAT, Pb 5’-FAM-CGG CGA ACT TCT TCT GCT AAT GGG GTT-BHQ1), *lcrE * (primer forward 5’-GAG GCT GTG TTG AGG TAG GT, primer reverse 5’-CGA TAA ATG CGG ATA ATG AGG AT, Pb 5’-FAM-AGG TAC TGG AGC ATG AGG AGG CGT A-RTQ1). RT-PR was performed on a CFX 96 amplifier (Bio-Rad Lab., United States). 

## RESULTS 

### Analysis of the structural similarity of the known 3TSS inhibitors 

Among the known 3TSS inhibitors [9–13], the compounds that belong to hydrazones based on hydrazides of aromatic carboxylic acids and various salicylic aldehydes (IV) have been the beststudied. The structures of T3SS inhibitors are shown in *Fig. 1* . 

These molecules are similar, to a certain extent, since all of them contain residues of salicylic and 4-hydroxybenzoic acids and their derivatives (these residues are shown in green). Also worthy of note is the structural similarity of the concatenation between the derivatives of salicylic acids and the remaining molecular part (shown in red). Based on the concept of bioisosteric replacement [14, 15], according to which the carbonylic and thiocarbonylic groups in biological systems are functionally interreplaceable, one can assume that the action of the thiohydrazones of oxamine acids and various salicylic aldehydes is similar to that of hydrazones. This class of compoundsis relatively new; until recently, its biological properties were almost completely outside the realm of study. 

Our goal was to resolve the following issues using thiohydrazones of thiohydrazides of oxamine acids as possible T3SS inhibitors: 

– obtain compounds with a low toxicity with respect to eukaryotic cells; 

**Table 1:** Toxicity indices of the selected compounds – thiohydrazones of thiohydrazides of oxamine acids

Compound №	Structure	Methylene blue staining, % of dead cells			Calcein assay, % of dead cells			MTT assay, % of metabolically inactive cells		
		12.5 µM	25 µM	50 µM	12.5 µM	25 µM	50 µM	12.5 µM	25 µM	50 µM
1	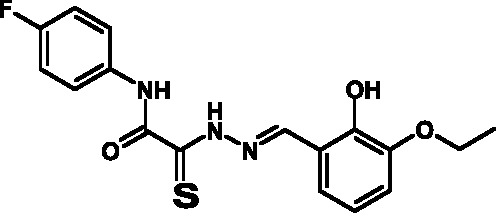	0+2	12+2	23+2	0+2	11+2	20+2	3+1	12+3	29+3
2	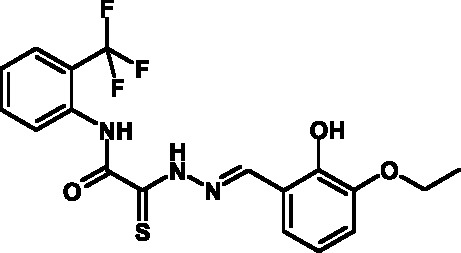	2+2	13+1	24+3	3+2	12+1	21+3	3+2	15+1	31+5
3	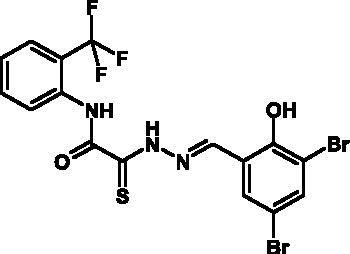	6+4	20+2	27+2	5+4	16+2	23+2	2+2	15+5	30+4
4	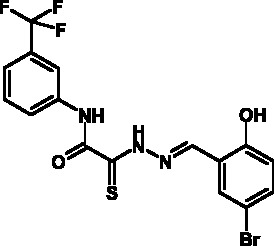	5+1	15+2	28+2	6+1	12+2	23+2	3+3	13+4	28+2
5	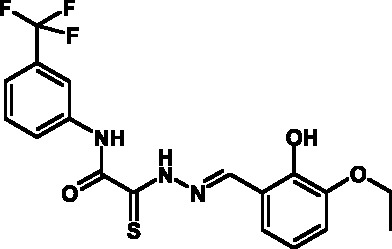	2+1	14+2	26+3	3+1	16+2	22+3	4+3	20+4	29+6
6	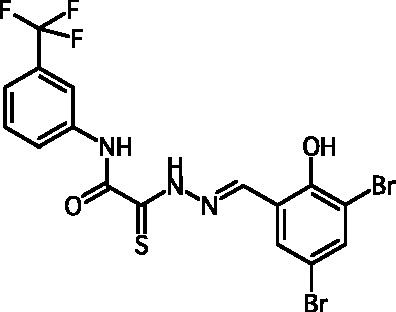	2+1	15+2	26+2	5+1	12+2	24+2	2+4	17+5	27+8
7	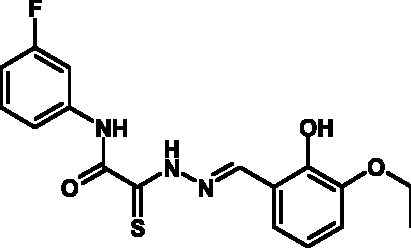	2+2	15+4	29+5	4+2	16+4	22+5	4+5	18+3	29+9
8	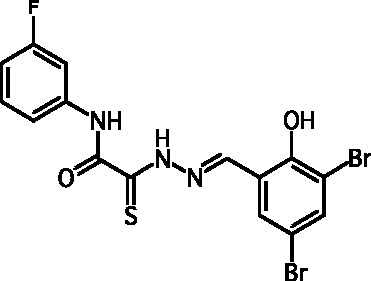	2+1	14+1	24+4	6+1	12+1	24+4	3+3	15+3	28+4
9	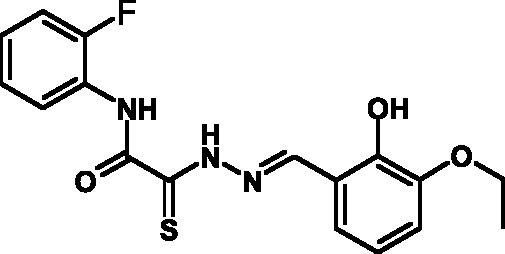	2+1	12+1	24+4	4+1	15+1	25+4	4+5	20+4	30+4
10	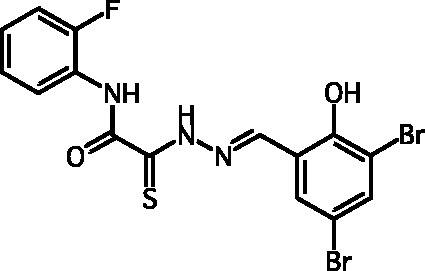	2+1	12+2	24+2	5+1	14+2	22+2	3+3	19+3	31+7
11	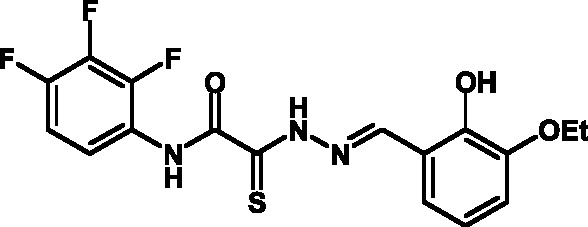	1+1	12+1	21+4	4+1	13+1	25+4	5+1	16+2	30+5
12	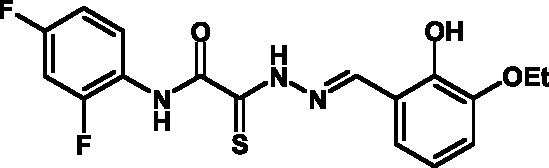	0+1	11+1	22+3	3+1	10+1	24+3	5+5	15+4	29+6
13	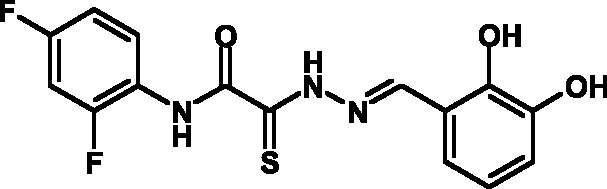	2+1	15+2	26+3	2+1	11+2	25+3	5+4	14+3	29+7
14	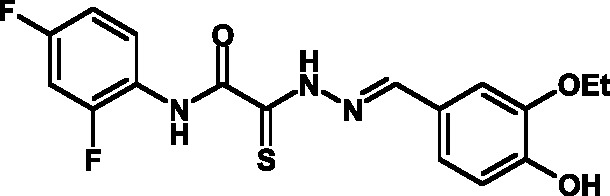	4+1	15+1	25+4	3+1	12+1	22+4	3+5	17+3	28+6
15	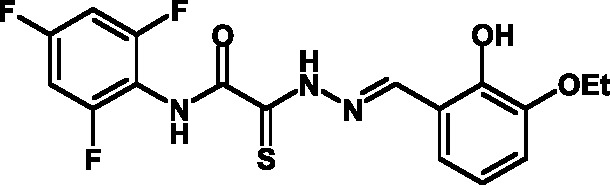	0+1	11+2	22+3	0+1	10+2	21+3	4+1	13+3	29+5

– obtain compounds with high selective activity with respect to T3SS; 

– obtain compounds with good pharmacokinetic properties; and 

–develop a simple scheme for the synthesis of the desired compounds. 

### Obtaining of thiohydrazones and testing their *in vitro* T3SS inhibition activity and toxicity  

**Fig. 3:**
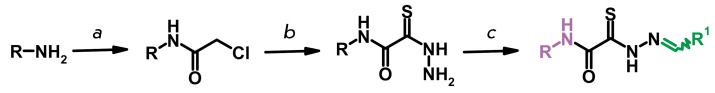
Synthesis of thiohydrazones based on the thiohydrazides of oxamineaicds: *a* – chloroacetyl chloride, DMF; *b* – 1) TEA, elemental sulphur, morpholine, DMF; 2) DMF, hydrazine hydrate; *c* – methanol, the corresponding aldehyde R ^1^ .

Thiohydrazones of oxamine acids ( *Fig. 2* ) were synthesized based on the appreciably simple scheme [16] shown in *Fig. 3* . 

It can be seen that a large variety of compounds can be obtained via the use of different commercially available amines and aldehydes via simple chemical conversions. A total of approximately 300 compounds have been synthesized; a number of compounds with good levels of solubility have been selected for further analysis. A total of 120 compounds were selected; despite having superior solubility as compared to that of hydrazones, thiohydrazonesgenerally have poor levels of solubility. 

**Fig. 4:**
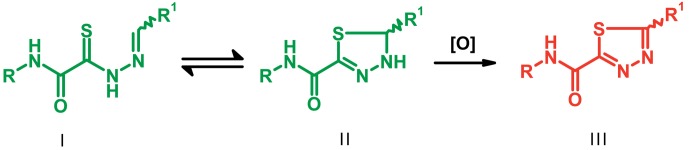
Ring-chain tautomerism of thiohydrazones and the oxidation product, thiadiazole.

**Table 2:** Inhibition of the intracellular development of *C. trachomatis* caused by the compounds under study

Compound №	Inhibition of the development of C. trachomatis (% of infection suppression) at different inhibitor concentrations, µM		
	12.5	25	50
1	30+4	99+7	100+1
2	70+5	98+3	100+1
3	1+1	30+6	60+5
4	5+2	10+5	60+11
5	0+1	10+3	75+6
6	20+4	40+5	90+12
7	15+3	85+8	100+2
8	5+3	10+5	90+13
9	30+8	40+7	90+10
10	0+2	10+7	98+3
11	10+2	90+7	100+1
12	0+1	70+15	100+3
13	15+5	70+14	100+15
14	15+4	45+7	100+3
15	40+3	85+8	100+2

The toxicity of the selected compounds for eukaryotic cells was assessed. First, the methylene blue staining technique was used. The compounds exhibiting satisfactory toxicity level were analyzed further using the calcein and MTT assays. As a result, 15 compounds were selected ( *Table 1* ). They manifested acceptable toxicity at concentration of 50 µM (death of less than 30% cells). All these compounds contained residues of various fluoroderivatives of aniline, as well as derivatives of salicylic and 4-hydroxybenzaldehyde. 

Chlamydial T3SS inhibitors are known to suppress *in vitro* the intracellular reproduction of the pathogen. It was shown, via testing of the ability of the selected compounds to suppress the chlamydial infection in the cell cultures, that all the compounds exhibit good inhibitory activity ( *Table 2* ). 

The ability of these compounds to suppress the translocation of *C. trachomatis * effector protein IncA was determined via immunofluorescence. It turned out that all the tested compounds inhibit the effector function of chlamydial T3SS. 

Thus, new inhibitors of *C. trachomatis * T3SS that belong to the class of thiohydrazones of the thiohydrazides of oxamine acids were selected; all the compounds suppressed Chlamydia reproduction in cell cultures. The presence of a fluorine atom can be assumed to enhance the lipophilicity of the selected molecules, which enables them to penetrate more easily through biological membranes, and presumably increases their resistance to various enzymes [17]. Moreover, the inclusion of the derivatives of salicylic and 4-hydrobenzaldehydes has enabled to obtain compounds with improved solubility and activity. 

### Investigation of the stability and the nature of the toxicity of thiohydrazones 

Based on the results of the analyses performed, three T3SS inhibitors were selected from a total of 15 for further study of their stability upon storage under various conditions. It turned out that these compounds in their dry form remained stable for a long time (according to the TLC data), whereas their activity in solutions decreased rapidly. 

It was ascertained via the analysis of the published data [18, 19] that ring–chain tautomerism is typical of thiohydrazones. Cyclic tautomers (thiadiazolines **II** ) are easily oxidized by air oxygen, yielding inactive and toxic thiadiazoles **III** ( *Fig. 4* ). 

**Fig. 5:**
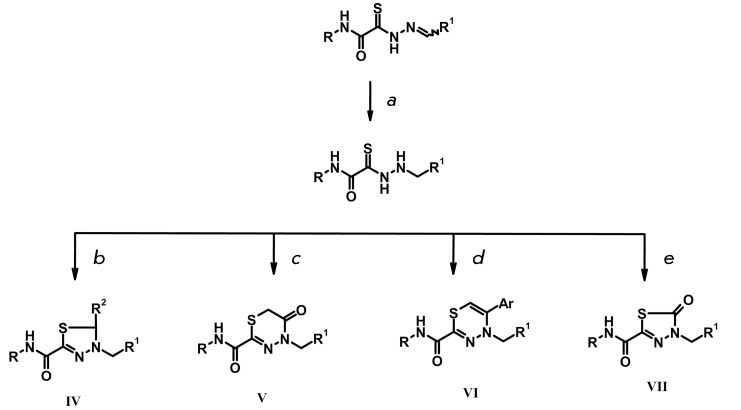
Synthesis of various heterocyclic compounds eliminating the existence of tautomerism and containing a closed thiocarbonyl group: *a* – sodium borohydride, methanol; *b* – aldehyde R ^2^ , isopropanol, hydrochloric acid; *c* –chloroacetic acid, isopropanol, ammonium acetate; *d* – ethanol, α-bromoketone, sodium acetate; *e* – carbonyldiimidazole, tetrahydrofuran.

These assumptions were experimentally verified via the synthesis of the hypotheticalthiadiazoles and by NMR studies of the thiohydrazones of oxamine acids. It was ascertained that the both cyclic and linear forms are indeed present in thiohydrazone solutions; the oxidation products in the solution are identical to the target-synthesized thiadiazoles. 

**Table 3:** Toxicity indices of thiadiazines

Compound №	Structure	Methylene blue staining, % of dead cells			Calcein assay, % of dead cells			MTT assay, % of metabolically inactive cells		
		12.5 µM	25 µM	50 µM	12.5 µM	25 µM	50 µM	12.5 µM	25 µM	50 µM
16	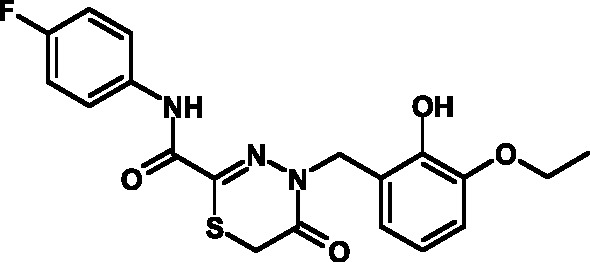	0+2	5+2	10+2	0+1	6+2	12+3	1+2	8+3	13+3
17	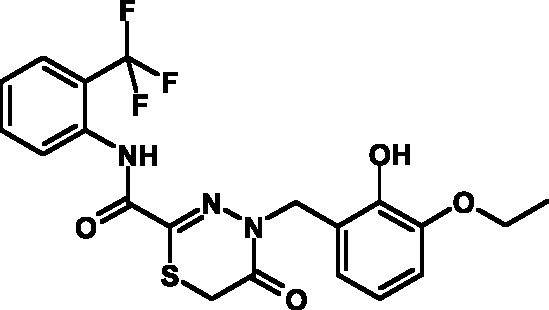	0+2	6+1	11+3	0+3	7+2	10+3	0+2	9+3	14+4
18	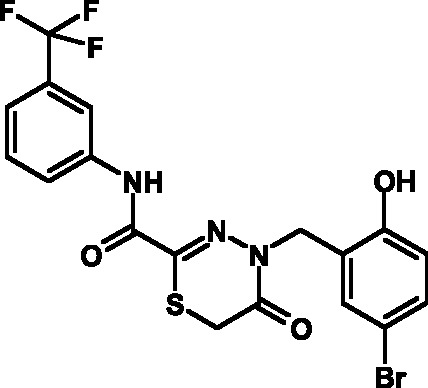	0+1	4+2	9+2	0+2	8+1	12+3	0+1	9+2	15+3
19	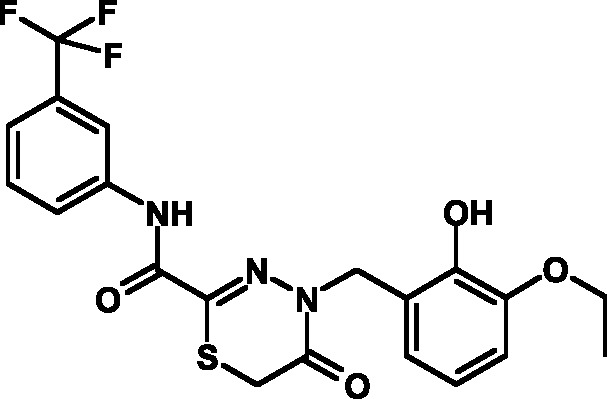	0+1	6+2	11+2	0+1	5+1	13+4	0+1	8+3	13+3
20	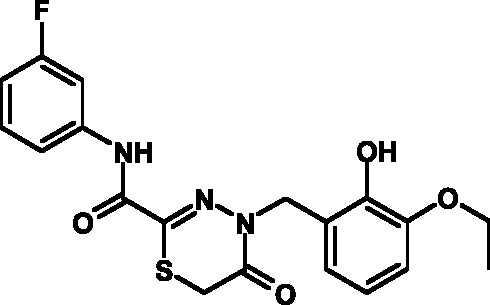	0+1	5+2	12+3	0+2	6+2	14+2	0+1	8+3	14+4
21	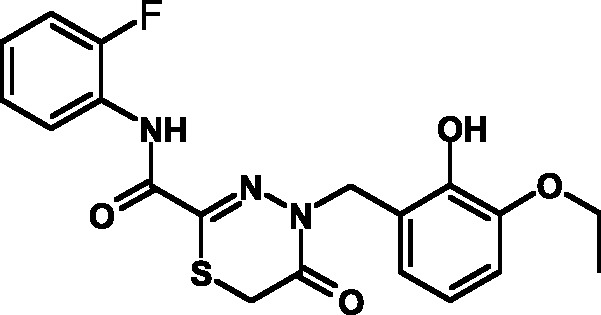	0+1	6+2	9+2	0+1	7+2	12+3	0+1	9+3	14+3
22	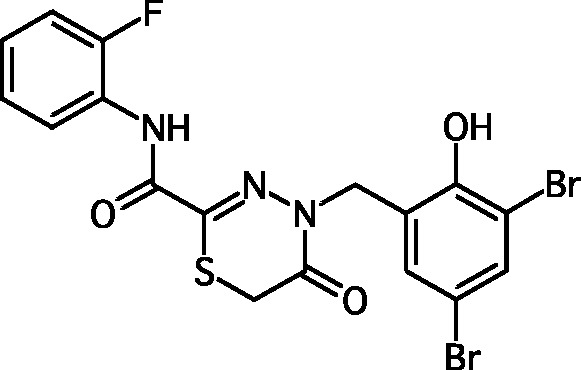	0+2	5+4	10+5	0+3	6+3	12+4	1+2	8+3	15+4
23	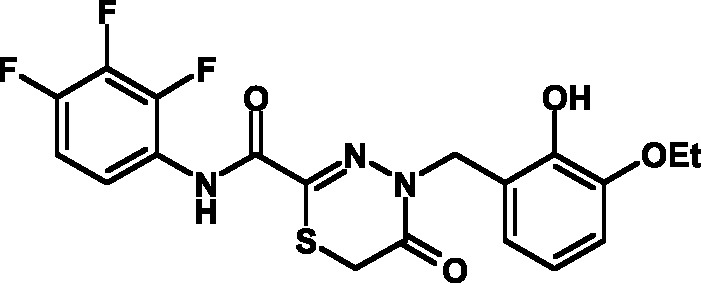	0+2	7+1	12+4	0+1	6+2	13+3	0+2	9+3	14+5
24	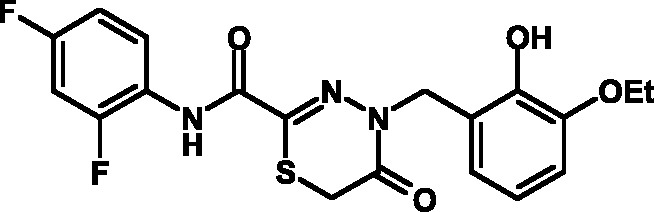	0+1	4+1	10+4	0+1	6+1	14+3	0+1	10+3	17+4
25	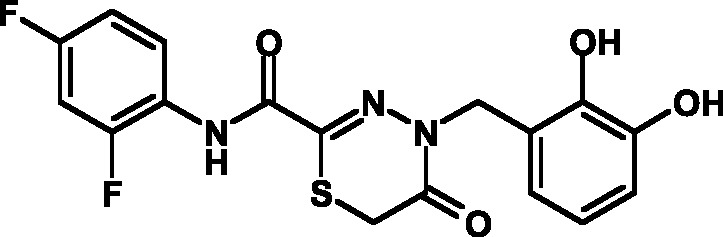	0+3	6+2	11+2	0+2	7+2	12+3	1+3	9+2	14+3
26	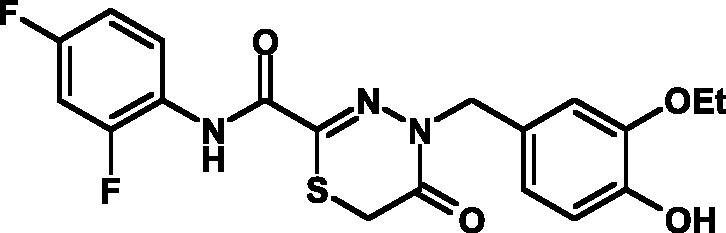	0+1	7+1	12+4	0+2	6+3	13+3	0+1	8+3	14+2
27	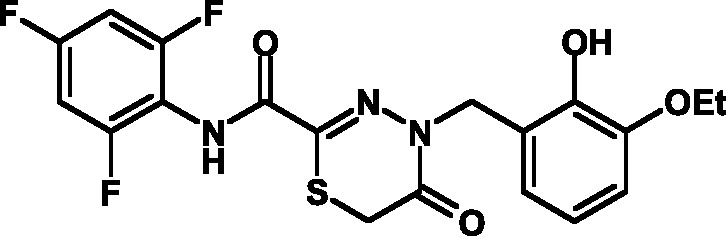	0+2	5+1	10+3	0+3	7+2	11+3	1+2	8+3	13+3

Attention was paid not only to the stability of the compounds, but also to the results of the MTT assay, which attested to an appreciably high toxicity of the compounds due to the suppression of the cellular respiration and the disruption of its oxidation-reduction potential. The analysis of the data published enables one to conclude that the open and considerably active thiocarbonyl group affects the level of toxicity; this group is bound to the reduced glutathione and induces the development of oxidative stress. This hypothesis was verified in the experiments in which glutathione was added to the cell culture medium, resulting in a 30%–40% decrease in toxicity. 

Thus, the task was to enhance the stability of the resulting compounds whilst reducing their toxicity in such a manner so as to prevent the activity and specificity from being adversely affected. 

### Modification of thiohydrazones aimed at enhancing stability and reducing toxicity 

In order to enhance the stability and reduce the toxicity of thiohydrazones, it was necessary to modify the structure so as to eliminate the formation of tautomeric forms and the open reactive thiocarbonyl group. 

This was achieved by means of the synthesis of several heterocyclic compounds based on reduced thiohydrazones, according to the diagram shown in *Fig. 5* . 

The synthesized heterocyclic derivatives of the thiohydrazones of oxamine acids were characterized by a higher solubility compared to the initial products; thus, their toxicity and capacity to inhibit T3SS was assessed. The compounds belonging to groups **IV** and **V** were characterized by low toxicity and exhibited specific activity with respect to T3SS, whereas the compounds belonging to groups **VI** and **VII ** did not exhibit such activity. 

Compounds belonging to group **IV** turned out to be instable in solutions (according to the TLC, the decomposition products were detected as early as 24 h after upon storage of the solutions at +20 ^o^ С). The compounds from group **V** were characterized by acceptable indicators. A total of 12 compounds belonging to group **V** were synthesized on the basis of the fluorine-containing thiohydrazones of oxamine acids. 

All these compounds proved considerably less toxic with respect to eukaryotic cells compared to the T3SS inhibitors that are already known and were obtained earlier ( *Table 3* ). 

It was demonstrated that the synthesized thiadiazinons suppress the development of intracellular infection in dose-dependent fashion. Four compounds at a concentration of 50 µM completely suppressed the infectious process in the cell culture ( *Table 4* ). The most efficient compound was selected via a comparison of the results of a determination of the toxicities and activities. This compound, known as CL-55, was used for a further, more advanced investigation of biological properties. 

### Inhibition of the effector function of T3SS by the synthesized inhibitor 

The specific activity of chemical compounds (i.e., their ability to inhibit the action of the type three secretion system (T3SS) was studied using the method based on the detection of the *C. trachomatis * effector protein. The IncA protein, one of the effector proteins of this pathogen, is synthesized in the bacterial cell, followed by secretion and incorporation into the chlamydial inclusion membrane. This protein is known to be synthesized 6 h following the onset of infection; it emerges at the inclusion surface after 8 h. Specific antibodies can be used to detect the IncA protein within the inclusion membrane. The inhibition of the translocation of the effector protein IncA of T3SS in *C. trachomatis* with a selected inhibitor is shown in *Fig* .  *6* . Small inclusions can be detected by cell staining with anti-C.trachomatis major outer membrane protein (anti-MOMP) antibodies; the size of the inclusions correlates with the infection period. 

**Table 4:** Inhibition of the development of *C. trachomatis* in a cell culture

Compound №	Inhibition of the development of C. trachomatis (% of infection suppression) at different inhibitor concentrations, µM		
	12.5	25	50
16	15+5	40+8	87+6
17	24+3	69+6	100+5
18	7+2	26+7	63+6
19	12+3	37+6	65+8
20	9+3	26+7	59+7
21	15+7	42+7	86+10
22	2+4	23+6	52+4
23	34+6	78+8	100+2
24	30+8	69+6	98+4
25	25+5	59+6	95+5
26	24+3	82+9	100+2
27	27+4	65+10	100+2

**Fig. 6:**
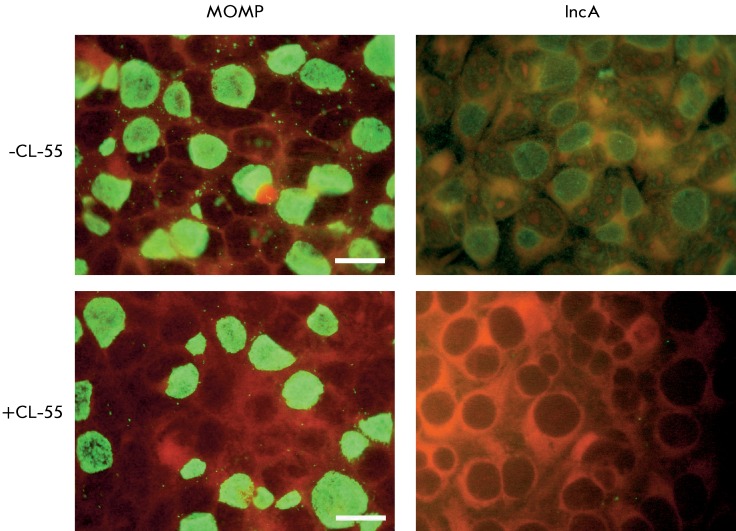
Inhibition of the translocation of the * C. trachomatis* effector protein IncA into the intracellular inclusion membrane under the action of the T3SS inhibitor, CL-55. The McCoy cells were infected with *C. trachomatis* ; 50 µM CL-55 was introduced into the culture medium after 8 h, and the medium was cultured for an additional 24 h. The chlamydial inclusions were stained with anti- *C. trachomatis* MOMP antibodies or anti-IncA antibodies and viewed under a luminescence microscope (green luminescence). Scale – 20 µM.

An additional assay enabling the assessment of the specificity of compounds with respect to T3SS is based on the fact that the chlamydial IncA protein translocated through T3SS participates in the fusion of separate inclusions that are developed inside a cell. Staining of the cells infected with anti-MOMP antibodies has demonstrated that the introduction of a compound affecting the T3SS 8 h following infection results in the formation of several small nonfused inclusions, whereas large inclusions (one per cell) were observed in the control cells ( *Fig. 6* ). 

### Effect of the T3SS inhibitor on the morphology of *C. trachomatis* intracellular inclusions 

The effect of the T3SS inhibitor, CL-55, on the intracellular development of the causative agent was studied by luminescence and electron microscopy. McCoy cell culture was infected with *C. trachomatis* and the inhibitor was simultaneously added to the culture medium at varying concentrations (12.5, 25 and 50 µM). Following 48 h, the cell cultures were analyzed by immunofluorescence and transmission electron microscopy. 

**Fig. 7:**
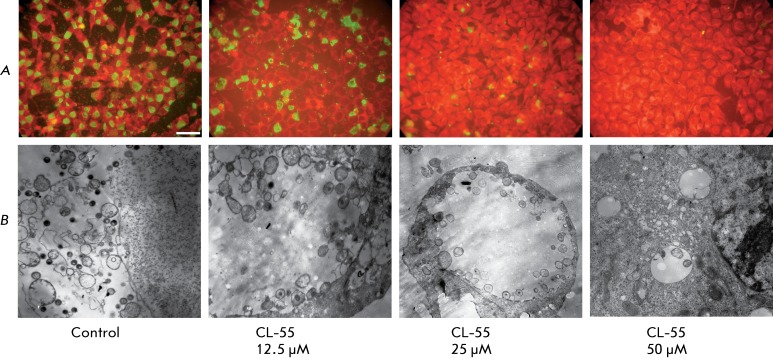
Dose-dependent inhibition of the intracellular development cycle of *C. trachomatis* under the effect of CL-55. *A* –luminescent microscopy after staining with anti- *C. trachomatis* MOMP antibodies. Scale – 10 µM. *B* – Transmission electron microscopy. Control x 4000, CL-55 (12,5 µM) x10000, CL-55 (25 µM) x 4000, CL-55 (50 µM) x10000

**Fig. 8:**
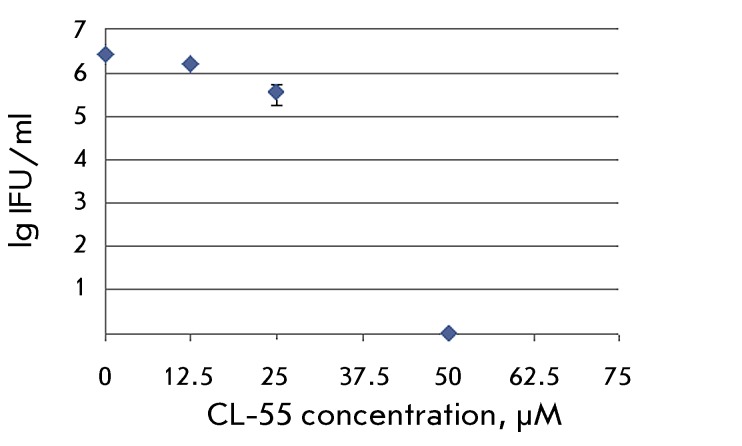
Suppression of the *C. trachomatis* viability after the addition of varying concentrations of CL-55.

The effect of CL-55 on the intracellular development of chlamydia resulted in the reduction of the count of infected cells and the inclusion size. At a dose as low as 12.5 µM, the inclusion count was 80% of the control ; the inclusions were smaller in this case. At CL-55 concentration of 25 µM, the accumulation of chlamydia was inhibited by 50%. The average size of inclusions was several times lower compared to that in the control specimen. Almost no inclusions were observed when using CL-55 at a dose of 50 µM ( *Fig. 7* ). 

A decrease in the inclusion size and a considerable decrease in the number of bacteria per inclusion were revealed through an electron microscopy study. Moreover, it should be noted that a typical pattern of termination of the chlamydial life cycle was observed in the control specimen: the overwhelming majority of the chlamydia inside the inclusions were of the extracellular form (the elementary bodies); some inclusions were disintegrated. Upon action of the T3SS inhibitor, the intracellular chlamydia was in the state of reticular bodies. Single, tiny inclusions containing abnormal bacterial cells were observed in the cells at a concentration of 50 µM. It has been thus demonstrated that the selected T3SS inhibitor dose-dependently suppresses the intracellular reproduction of *C. trachomatis.*

### Effect of CL-55 on *C. trachomatis * progeny 

The effect of the T3SS inhibitor on chlamydial development was also assessed using the semi-quantitative method for the *in vitro * determination of the infectious properties of the pathogen. The calculation of the count of *C. trachomatis * infectious particles after the action of the inhibitor at different concentrations demonstrated a dose-dependent reduction ( *Fig. 8* ) and a complete viability inhibition in the presence of 50 µM of CL-55. Meanwhile, a significant number of the inclusions being formed possessed an atypical morphology at lower concentrations of the compound. No intracellular inclusions were formed upon further passaging. 

### Effect of CL-55 on gene expression in *C. trachomatis *

The effect of the T3SS inhibitor on the activity of constitutive genes, the 16S rRNA and *trpA* , the tryptophan operon gene, and on the expression of the *incA* gene encoding the synthesis of the effector protein, and the *lcrE* gene of the T3SS regulatory protein in *C. trachomatis* were studied at this stage. The inhibitor was introduced simultaneously with cell infection; after 24 h, the RNA was isolated and the gene expression was analyzed using quantitative real-time PCR. 

The use of the compound at concentrations of 25 and 50 µM reduced the activity of the 16S rRNA gene by 4 and 29 times, respectively. All specimens were subsequently normalized with respect to cDNA of the 16S rRNA DNA. The activity of the *trpA* and *incA* genes under action of 25 and 50 µM CL-55 remained unchanged compared to the control. These data attest to the following facts:firstly, the inhibitor had no pronounced effect on the pathogen metabolism; secondly, the inhibitor had no effect on the expression of the effector protein IncA gene at transcriptional level. The expression of the *lcrE* gene encoding the T3SS regulatory protein reduced by 90% in the presence of 50 µM. The study of the effect of CL-55 on the expression of T3SS-specific chlamydial genes aimed at elucidating the mechanism of action of the selected inhibitor wastaken further recently. 

### Effect of the inhibitor CL-55 on the intracellular development of other chlamydial species 

The effect of the selected T3SS inhibitor on the intracellular reproduction of representatives of two other species belonging to the *Chlamydiaceae* family, *C. pneumoniae * and *C. muridarum* , was assessed using the methods described above. The fact that CL-55 at a concentration of 50 µM completely inhibited both the intracellular accumulation and the viability of these chlamydial species attested to the universality of the effect of this inhibitor on the other Chlamydia. 

## DISCUSSION 

The type III secretion system detected only in pathogenic bacteria was selected for use as a target for the search for new antibacterial drugs that would demonstrate efficacy with respect to both acute and chronic infections. The formation of this secretory apparatus, the so-called “molecular syringe,” starts after contact with a eukaryotic cell. It forms a pore in the target cell membrane, resulting in the direct penetration of the pathogenicity factors into the host cell’s cytoplasm [20]. T3SS can also function upon intracellular localization of the pathogen. The transport of the pathogenic factors results in cytoskeletal rearrangement, apoptosis inhibition, modification of the apparatus of transcription and translation in the eukaryotic cells, modulation of cytokine production and other processes in the host cell which facilitates pathogen invasion, blockage of host protection, and the establishment of continuous persistence [21]. T3SS is absolutely essential for the development of an acute infectious process; the chronization of the infection fundamentally depends on how it functions. Thus, the specific inhibition of the T3SS function is expected to interrupt the infectious process both at early stages and upon a chronic course, thus allowing the immune system to eliminate the pathogen. 

New efficacious T3SS inhibitors were explored according to the following scheme: similar regions were found in the molecules of organic compounds via a structural analysis of the known T3SS inhibitors, thereby enabling the construction of a new class of compounds exhibiting antibacterial activity that is specific with respect to T3SS. A significant number of such compounds have been synthesized, enabling molecular screening using cellular assays and with the selection of 15 compounds that specifically inhibit chlamydial T3SS *in vitro* in order to study the structure–activity relationship. The selected compounds were subsequently modified in order to enhance their solubility, stability, and biological activity, to reduce toxicity for eukaryotic cells, and enhance specific efficiency. 

All this enabled to obtain a new T3SS inhibitor belonging to the class of heterocyclic compounds. This low-molecular-weight compound blocked the effector function of *C. trachomatis * T3SS. Thus, IncA (one of the early effector T3SS proteins) was not detected on the membrane of the chlamydial inclusion, and the process of homotypic phagosome fusion mediated by this protein was disrupted with the introduction of CL-55. An analysis of the expression of the *incA* gene has shown that the inhibitor does not reduce the transcription of this gene. A conclusion can thus be drawn that the selected compound is capable of selective inhibition of the translocation of the chlamydial effector protein. 

Compound CL-55 had no pronounced inhibiting action on the level of expression of the *C. trachomatis* constitutive genes, a fact in agreement with the known mechanism of action of the T3SS inhibitors. According to this mechanism, the inhibitor affects the functioning of the secretory apparatus rather than the metabolism of a bacterial cell. Meanwhile, a considerable reduction of gene activity in one of the key regulators of chlamydial T3SS (CopN protein) was observed. Under normal conditions, this gene is expressed at all stages of the intracellular life cycle of chlamydia. Functioning as a chaperon protein, it participates in the secretion regulation of the proteins of the Inc family at the early stages of intracellular development and regulates the T3SS-mediated differentiation of reticular chlamydial bodies into elementary bodies at the late stage of life cycle. It is of importance that the proliferation of reticular bodies depends on direct contact with an inclusion membrane and on interaction with the effector proteins translocated into it. Being a negative regulator, the CopN protein reduces the translocation of the major effector proteins (including IncA) on the inclusion membrane and closes the channel, thus impeding the translocation of the other T3SS effectors [22]. The resulting decrease in the expression of the gene encoding this regulatory protein can be an indicator of the specific action of the selected inhibitor on the regulation of the function of the chlamydial T3SS. The effect of the CL-55 inhibitor on the activity of a number of genes regulating the activity of *C. trachomatis * T3SS is currently under investigation. 

Since the functioning of the chlamydial T3SS determines the possibility of intracellular development of the pathogen, the specific T3SS inhibitor is intended to disrupt the life cycle and block the infectious process upon both acute and persistent forms of the infection. The designed inhibitor suppressed the reproduction of three chlamydial species, *C. trachomatis, C. pneumoniae,* and *C. muridarum* , on cell culture models. The suppression manifested itself in the following ways: in the changes observed in the morphology of the chlamydial inclusions, by the disruption in the differentiation of the reticular bodies into elementary bodies, and by the inhibition of the infectious properties of the pathogen. Moreover, it has been demonstrated (the results are not shown) that the selected inhibitor blocked the secretion of the T3SS effector proteins in Salmonella, a representative of the taxonomically non-related group of pathogenic bacteria, which may attest to the universality of the resulting T3SS inhibitor. Meanwhile, the compound exhibited no bactericidal effect on a number of Gram-negative and Gram-positive bacteria, representatives of the normal bacteria.

Thus, a new T3SS inhibitor belonging to the class of heterocyclic compounds has been obtained via targeted chemical synthesis, experimental screening, and chemical optimization. This compound is currently being studied with the use of experimental animals for its therapeutical activity and pharmacokinetic properties. These studies are intended at probing the application of the compound for the further development of an antibacterial drug that would be efficacious with respect to the acute and chronic forms of infection.  

## Abbreviations

T3SS: type III secretion system
MOI: multiplicity of infection
МОМР: major outer membrane protein of Chlamydia
LPS: lipopolysaccharide
EB: elementary bodies
RB: reticular cells
IFU: inclusion forming units

## References

[1] Shiinsky G.E., Merzlyakov V.A., Timofeeva S.B. (1999). Vestn. dermatologii i venereologii..

[2] Dean D. (2009). Drugs Today (Barc.)..

[3] Bashmakov Y.K., Zigangirova N.A., Pashko Y.P., Kapotina L.N., Petyaev I.M. (2010). Comp. Hepatol..

[4] Zigangirova N.A. (2009). Sb. nauchnikh trudov: &quot;.

[5] Zigangirova N.A., Fedina E.D., Zorinа V.V., Bortsov P.A. (2009). Journal Microbiology and Epidemiology and Immunobiology..

[6] Guntsburg A.L., Zigangirova N.A., Zorina V.V. (2008). Vestn Ross Akad Med Nauk..

[7] Erhardt M., Namba K., Hughes K.T. (2010). Cold Spring Harb Perspect Biol..

[8] Karyagina A.S., Alexeevsky A.V., Spirin S.A., Zigangirova N.A. (2009). Molecular Biology..

[9] Felise H.B., Nguyen H.V., Pfuetzner R.A., Barry K.C., Jackson S.R., Blanc M.P., Bronstein P.A., Kline T., Miller S.I. (2008). Cell Host & Microbe..

[10] Baileya L., Gylfe A., Sundin C., Muschiol S., Elofsson M., Nordström P., Henriques-Normark B., Lugert R., Waldenström A., Wolf-Watz H. (2007). FEBS Lett..

[11] Tautz L., Bruckner S., Sareth S., Alonso A., Becattini B., Salvesen G.S., Mustelin T. (2005). J. Biol. Сhem..

[12] Kauppi A.M., Andersson C.D., Norberg H.A., Sundin C., Linusson A., Elofsson M. (2007). Bioorg. Med. Chem..

[13] Kauppi A.M., Nordfelth R., Uvell H., Wolf-Watz H., Elofsson M. (2003). Chem. Biol..

[14] Patani G.A., LaVoie E.J. (1996). Chem Rev..

[15] Yang H., Hendricks R.T., Arora N., Nitzan D., Yee C., Lucas M.C., Yang Y., Fung A., Rajyaguru S., Harris S.F. (2010). Bioorg. Med. Chem. Lett..

[16] Gunzburg A.L., Zigangirova N.A., Tokarska E.A., Zorina V.V. Patent №2400471 27.09.2010 г. RF. С07D 213/75,
 С07D 338/38, С07С 327/56,
С07С/225/16..

[17] Hagmann W.K. (2008). J. Med. Chem..

[18] Zelenin K.N., Khrustalev V.A., Alekseev V., Sharbatyan P.A., Lebedev A.T. (1982). Khimia geterotsikl. soed..

[19] Lempert-Sreter M., Lempert K., Möller J. (1983). J. Chem. Soc. Perkin Trans..

[20] Cornelis G.R. (2006). Nat. Rev. Microbiol..

[21] Hoare A., Timms P., Bavoil P.M., Wilson D.P. (2008). BMC Microbiol..

[22] Delphine S., Beeckman A., Daisy C., Vanrompay G. (2010). Curr. Issues Mol. Biol..

